# Factors Associated with Perceived Life Chaos among Post-Myocardial Infarction Survivors in a Malaysian Cardiac Care Facility

**DOI:** 10.3390/medicina54050079

**Published:** 2018-11-05

**Authors:** Kurubaran Ganasegeran, Abdul Rashid

**Affiliations:** 1 Department of Public Health Medicine, RCSI-UCD Malaysia Campus, 4 Sepoy Lines, Penang 10450, Malaysia; rashid@pmc.edu.my; 2 Clinical Research Center, Seberang Jaya Hospital Ministry of Health Malaysia, Jalan Tun Hussein Onn, Seberang Jaya, Penang 13700, Malaysia

**Keywords:** life chaos, social support, myocardial infarction, survivors, Malaysia

## Abstract

*Background and objectives:* Survivors of chronic life-threatening conditions like myocardial infarction (MI) are often confronted with multiple physical and psychological stressors as a consequence of elevated demands of lifestyle adjustments and modifications. Such stressors, collectively known as “life chaos”, cause disruption to one’s lifestyle equilibrium of having organized, calm, and regular routines. The objective of the current study was to determine the level of life chaos and its associated correlates among post-myocardial infarction (post-MI) survivors in Malaysia. *Materials and Methods:* An analytical cross-sectional study was conducted among 242 post-MI survivors in a Malaysian cardiac health facility from July to September 2016. A self-administered questionnaire in Malay that consisted of items on socio-demographics, health attributes, validated OSLO-3 Social Support Scale (OSS-3), and the Modified Confusion, Hubbub, and Order Scale (CHAOS-6) was utilized in this study. Descriptive, bivariate, and multivariate analyses were conducted. *Results:* The sample constituted of 208 (86%) men and 34 (14%) women. The average age was 55 years (SD = 11), and the age ranged between 24 and 96 years. Overall, 128 (52.9%) of the total post-MI survivors had highly chaotic lives. In multivariate analysis, younger age, lower household income, perceived financial insecurity, poor health status, and multiple comorbidities were related to the high chaos score, and these associations were statistically significant (*p* < 0.05). *Conclusions:* Highly chaotic lifestyles were prevalent in post-MI survivors. Demographic, health attributes, and socio-economic factors were important correlates of life chaos.

## 1. Introduction

*Chaos often breeds life, when order breeds habit.*

The Education of Henry Adams; 1907/1918

Ancient theorists often conceptualized the importance of the environment on health and well-being. They postulated that the locus of healing within an individual is optimized when calmness is offered within the best supportive and environmental conditions to allow “nature” to act upon them [1]. Empirical studies that explored phenomenal interactions between “atmosphere” and “environment” concluded that psychosocial and physical surroundings were inseparable entities that optimize the process for recuperation among people with chronic illnesses [2].

With the population’s health research paradigm shifting from disease to risk, the concept of life chaos has emerged in recent literature as a potential novel psychological attribute that influences human well-being [3,4]. Chaos has been defined as an overall physical, social, and environmental disorder in a person’s life, constituting high levels of confusion, agitation, sense of rush, being disorganized, and unable to cope with daily routines [5]. 

Rapid urbanization, poverty, poor housing, noise, pollution, and poor neighborhood integration that inhibits structural social support has catalyzed negative consequences to people’s lifestyle transformation (PLT) [6,7]. In addition, people who have survived chronic illnesses like myocardial infarction (MI) are often confronted with multiple physical and psychological stressors, including unemployment, homelessness, rapid socio-financial decline, reduced social support, and deteriorating mental health status, which disrupts one’s ability to engage in therapeutic lifestyle changes (TLC) for better quality of life [4]. Contemplating sickness outcome, chaos may elicit externalizing behavior problems that aggravate anger or aggressiveness due to elevated frustrations of physical and functional disabilities [8]. In contrast, chaotic people may also capitulate to deleterious effects of internalizing behaviors like anxiety withdrawal due to the emotional shock of the diagnosis [9]. 

Chaotic lifestyles may disrupt one’s ability to be compliant with appropriate lifestyle modifications and adjustments, predisposing patients to move away from continued medical care and engage in risky behaviors, missed appointments, or adhere poorly to agreed treatment plans [4,10]. In a study conducted among HIV-infected adults in Los Angeles, Wong et al. [3] found that life chaos was more prevalent among persons with risky health behaviors, homelessness, or unmet needs. Studies in the United States of America and Malaysia found an association between life chaos and adherence to treatment recommendations [4,10,11]. 

Cardiovascular diseases (CVDs) cause unprecedented mortality and disabilities worldwide, with resource-poor and middle-income countries suffering the bulk of the total CVDs epidemic. In Malaysia, CVDs—particularly MI and its related complications—have topped the nation’s mortality rate, followed by deteriorating mental health outcomes [12]. With elevated anxiety and concerns, survivors of myocardial infarction and their families are often helpless in the face of new demands, adaptation, and changes in their life routines to cope with consequences of the illness [13]. These vulnerabilities influence the coping mechanisms, resulting in disarray in family routines [13]. Based on the literature, we hypothesize that the lives of post-MI survivors are disorganized and chaotic as a consequence of MI. The current study aims to determine the level of life chaos and its associated factors among post-MI survivors in Malaysia.

## 2. Materials and Methods

### 2.1. Study Setting and Population

An analytical, hospital-based, cross-sectional study was conducted between July and September 2016 amongst 261 post-MI survivors at the Cardiology Outpatient Clinic of Serdang Hospital, Selangor, Malaysia. Being one of the three premier cardiac referral centers in Malaysia (apart from the National Heart Institute in Kuala Lumpur and the Sarawak General Hospital in Borneo), the hospital shares an equal burden for cardiac referral cases throughout Peninsular Malaysia [14].

### 2.2. Sample Size Determination

With a population of over 500 post-MI survivors from Serdang Hospital between January and December 2015 [15], a sample size of 217 patients was calculated to allow the study to determine the prevalence of life chaos with a confidence interval of ±5% [16]. An additional 20% were included in the calculated sample [17] to compensate for possible missing data or nonresponse. As a result, a final sample size of 261 was determined as adequate to achieve the objectives of the study. 

### 2.3. Participant Recruitment

Participants were selected using a systematic sampling method, where every third patient attending the myocardial infarction clinic was selected. Eligibility of the sample recruitment included confirmed post-MI survivors as diagnosed by a physician and documented in medical records, aged 18 years or older, and discharged from the hospital at least one month post-MI. Patients who were cognitively impaired, under treatment for active mental illness, or unable to read or comprehend in Malay were excluded from the study.

### 2.4. Ethical Consideration

This study complied with the guidelines convened in the Declaration of Helsinki. Ethical approval was obtained from the Medical Research Ethics Committee (MREC), Ministry of Health Malaysia (government approval number: NMRR-15-2210-28696-IIR, date of approval 11 April 2016). The aim and objectives of the study was explained to the respondents. Confidentiality, anonymity, and their right to withdraw from the study were assured. Participants received a written description of the purpose, as well as aims and benefits of the study, along with the study questionnaires in a sealed envelope. Participant consent was obtained from those who agreed to participate. 

### 2.5. Study Instrument

Based on literature and validated scales, a self-administered questionnaire that consisted of four parts was developed in the local language (Malay).

The first part consisted of information related to socio-demographic variables (gender, age, marital status, household income, current employment status, and perceived financial insecurity). 

In the second part of the questionnaire, information related to health attributes was obtained. Self-rated general health was assessed using a single validated item “How would you rate your current health status?” with five response options: poor (5), fair (4), good (3), very good (2), and excellent (1) [18]. These items were reversed and dichotomized as good (1–3) and poor (4–5), consistent with previously reported scoring rule [19]. Disease comorbidities (diabetes, hypertension, or hypercholesterolemia) were derived from patient records. The baseline clinical parameters adopted were as follows: (1) Patients with fasting plasma glucose level of 7 mmol/L (126 mg/dL) or above and prescribed with oral hypoglycaemic agents or insulin regimen as documented in medical records were classified as having diabetes [20]. (2) Patients were recruited as hypertensive if they were previously diagnosed with hypertension and administered with antihypertensives as documented in medical records [20]. (3) Hypercholesterolemia was defined as total cholesterol of more than 5.2 mmol/L with high plasma triglyceride concentration (>1.7 mmol/L), low high-density lipoprotein (HDL) cholesterol concentration (<1.0 mmol/L for men; <1.3 mmol/L for women), and increased concentration of low-density lipoprotein (LDL) cholesterol (>2.6 mmol/L with cardiac risk factors) with patients currently on statins as documented in medical records [21]. Disease complications (diagnosis of atrial fibrillation and symptoms of heart failure) were derived from patient medical records.

In the third part, we administered three items that assessed social support, adapted from the validated OSLO-3 Social Support Scale (OSS-3) [22]. This brief measure, which can be used as an item-by-item scale, measures three main aspects of social support: number of people the respondents feel close to (structural support), the interest and concern shown by others (emotional support), and the ease of obtaining practical help from others (practical support) [23].

The primary outcome that measured chaotic lifestyles was assessed in the fourth part. The recently validated Malaysian version of the six-item Modified Confusion, Hubbub, and Order Scale (CHAOS-6) was administered to determine universal level of chaos in the sample [24]. The items that were consistent with the brief CHAOS-6 utilized by Wong and colleagues [3] to measure life chaos in HIV adults was extracted from the original 15-item CHAOS scale developed by Matheny and colleagues [25]. This six-item scale had the following items: “My life is organized”; “My life is unstable”; “My routine is the same from week to week”; “My daily activities from week to week are unpredictable”; “Keeping a schedule is difficult for me”; and “I don’t know what might come up”. Response choices were recorded on a five-point Likert scale ranging from “definitely true” to “definitely false”. Several questions were reverse-coded (all except first and third items) as these statements were negatively worded. The exploratory factor analyses yielded one factor with given Eigenvalues greater than 1 (3.0). The one-factor solution accounted for 50.0% of the variance. Factor loading ranged from 0.55 to 0.79. Cronbach’s alpha coefficient of the Confusion, Hubbub, and Order Scale (CHAOS-6) was 0.80. Scoring of the CHAOS-6 was performed by summing item responses, which ranged from 6 to 30, such that higher scores represented a more chaotic lifestyle [3,4,24]. As the data was normally distributed and to ensure real-life interpretations to determine the prevalence of life chaos in the sample, the total score based on mean cut-off points was dichotomized (a score of less than 15 representing a low chaotic lifestyle and a score of 15 or more representing highly chaotic lifestyles). 

### 2.6. Statistical Analyses

Analysis was performed using IBM SPSS Version 23.0 statistical software. Descriptive analysis was performed for all variables in this study. To assess the validity of CHAOS-6 for the study sample, an exploratory factor analysis was performed with varimax rotation, and Cronbach’s alpha was used to test the internal consistency of the scale. Continuous variables and total chaos score was expressed in mean and standard deviations (SDs). Test of normality was performed for total chaos score. Student *t*-test and analysis of variance (ANOVA) were applied to compare life chaos with socio-demographics, health attributes, and social support variables. In case of ANOVA, post-hoc Bonferroni test was used to determine where the statistically significant difference was. The primary outcome measure was maintained as a continuous variable in determining bivariate associations in line with the scoring methods of the original scale [3,4] and to ensure more power to the results [26] without deliberately discarding data via categorization of variables that could alarm false positives [27]. Multiple linear regression analysis using “backward elimination” technique was employed to obtain factors significantly associated with life chaos among post-MI survivors. The accepted level of statistical significance was set below 5% (*p* < 0.05). Multicollinearity was checked between independent variables. 

## 3. Results

### 3.1. Sample Characteristics

The data of 242 respondents (93% response rate) were included in the final analysis (19 questionnaires were excluded due to missing data and nonresponse). Of the total, 208 (86%) were men and 34 (14%) were women. The average age was 55 years (SD = 11), and the age ranged between 24 and 96 years. Most respondents (208; 86%) were married, not working (132; 54.5%) at the time of the study with a monthly household income between MYR 1001 and 3000 (143; 60.6%). The bulk of respondents perceived to be financially insecure (161; 66.5%) (Table 1).

### 3.2. Clinical Health Characteristics and Social Support of the Respondents

Baseline clinical data are exhibited in Table 2. Most respondents perceived good health status (73.1%). Of the total post-MI patients, 154 (63.6%) had two or more comorbid conditions; 115 (47.5%) suffered diabetes, 164 (67.8%) had hypertension, and 152 (62.8%) were diagnosed with hypercholesterolemia. With regard to social support, most respondents had very easy or easy access to instrumental social support (128; 52.9%). Majority (110; 45.5%) of the respondents perceived a lot of concern from others. Ninety (37.2%) respondents had 3–5 persons to count on in times of trouble. 

A total of 128 respondents (52.9%) perceived their life to be highly chaotic. The mean (SD) of the total CHAOS score was 15.0 (5.3), and the scores ranged from 6 to 30 (Table 2).

### 3.3. Association between Life Chaos and Sample Characteristics

Table 3 shows the association between life chaos and sample characteristics. Patients aged between 18 to 59 years had higher life chaos score (15.7 ± 5.4) compared to those aged 60 years or older (13.8 ± 4.8, *p* = 0.005). A statistically significant association was observed between life chaos and monthly household income (*p* = 0.027); post-hoc tests showed that those with an income level of MYR 1000 or less exhibited greater chaos (16.7 ± 6.1) in comparison to those with an income level of MYR 3001 or more (13.1 ± 4.3, *p* = 0.031). Patients perceived to be financially insecure were more chaotic (16.0 ± 5.2) in comparison to those who were not (13.2 ± 4.9, *p* < 0.001).

### 3.4. Association of Life Chaos with Health Attributes and Social Support 

Table 4 exhibits the association of life chaos with health attributes and social support. Patients who perceived poor health status had higher chaos score (17.3 ± 5.7) compared to those who perceived good health status (14.2 ± 4.8, *p* < 0.001). Patients with diabetes had higher chaos score (15.8 ± 5.3) in comparison to those without such condition (14.3 ± 5.1, *p* = 0.029). Those being diagnosed with two or more disease comorbidities had higher chaos score (15.8 ± 5.2) in comparison to those with less than two comorbid conditions (13.8 ± 5.2, *p* = 0.005). Significant associations were observed between chaos and concerns showed by others (*p* = 0.029); post-hoc tests revealed that patients who responded a little/none/uncertain regarding concerns shown by others had greater chaos score (16.5 ± 5.0) compared to those who had a lot of concern shown by others (14.2 ± 4.9, *p* = 0.032).

### 3.5. Factors Associated with Life Chaos among Post-MI Patients by Multiple Linear Regression

Table 5 shows the factors associated with life chaos among post-MI patients. Patients aged 18–59 years had on average 2.0 (95% CI 0.6 to 3.3) higher chaos score in comparison to those aged 60 years or older (*p* = 0.004). Those with monthly household income of MYR 1000 or less had on average 1.8 (95% CI 0.2 to 3.4) higher chaos score compared to those with income level of MYR 3001 or more (*p* = 0.026). Those perceived to be financially insecure had on average 1.9 (95% CI 0.6 to 3.3) greater chaos score in comparison to those who were not (*p* = 0.006). Similarly, patients perceived to have poor health status had on average 1.9 (95% CI 0.4 to 3.4) higher chaos score compared to those perceived in good health (*p* = 0.013). Patients with two or more disease comorbidities had on average 1.7 (95% CI 0.3 to 3.0) greater chaos score compared to those with less than two comorbid conditions (*p* = 0.014). The total model was significant (*p* < 0.001) and accounted for 21% of the variance. There was no multicollinearity between variables.

## 4. Discussion

### 4.1. How Chaotic Are Post-MI Survivors?

The present study showed that more than half (52.9%) of the Malaysian post-MI survivors perceived their lives to be highly chaotic (based on the cut-off point of ≥15). No published study conducted in Asia was available for comparison. At present, this is the first study to explore life chaos among post-MI survivors in Asia, especially in Malaysia. The relatively limited literature that has explored life chaos in adults has mainly focused on western communities. To date, three studies have determined life chaos among adults living with HIV [3,28,29], two studies have explored life chaos in American MI cohorts [4,10], one study has utilized qualitative approach to understand the process of “living in chaos and striving for control” among attention deficit hyperactivity disorder (ADHD) adults [30], and another investigation has studied life chaos among methamphetamine-using men who have sex with men (MSM) and engage in transactional sex [31]. All these studies showed high levels of chaotic lifestyles, which is consistent with the findings of the current study. 

### 4.2. What Factors Relate to Chaotic Lifestyles?

The results indicated that younger-aged survivors were more chaotic compared to older-aged survivors. This finding is contrary to previous studies among post-MI patients [4] and HIV-infected persons [3], which found no significant associations between age and life chaos. It could be argued that older aged people are more likely to be disorganized due to higher risks of impaired cognitive functions, decline in physical and physiological functions, and lower levels of education; these factors all reduce control in their lives, risking them to greater degree of chaos [10]. However, young adults who have greater employment, financial, social, or family engagements may have elevated frustrations and fear as a consequence of MI due to the high demands of therapeutic lifestyle adjustments, impacting their ability to be inclined with their previous job and threatening potential financial instability, greater debts, and disorganization within the family institution, which can potentially make life more disorganized [10,32]. 

Lower socio-economic status predisposes post-MI survivors to live in worse physical conditions, such as having greater exposure to noise or overcrowding, and may lead to health-compromising behaviors like being nonadherent to agreed treatment plans [10,32]. This study found that survivors with lower income level and those perceived to be financially insecure showed greater life chaos. These findings were consistent with previous studies [3,4,29]. As a result of multiple unanticipated competing demands from lower socio-economic status, post-MI survivors are faced with greater unpredictability in their lives and, thus, unlikely to experience stability in their daily routines due to limited resources for addressing individual or family needs. 

Two potential novel associations were exhibited in our study: (1) Survivors who perceived poor health status and those suffering two or more disease comorbidities showed greater chaos score. (2) These associations were statistically significant. The plausibility of these significant associations could be interpreted with the theoretical concept postulated by Bury [33], which advocated chronic life-threatening illnesses as “biographically disruptive”. Chronic illnesses like MI leads to a loss of independence and possibly an economically nonproductive life. The feeling of worthlessness and loss of sense of oneself due to demands of lifestyle adjustments after MI may predispose survivors to chaotic lifestyles and discontinuity of normal routines [9]. 

The bulk of the literature have highlighted the importance of social support in post-MI survivors; having fragmented or fragile social support networks inhibits fundamental coping mechanisms to overcome unexpected psychosocial repercussions like chronic stress, fast-paced life, having too much responsibilities, accumulation of unpredicted life events, depression, anxiety, social isolation, and withdrawal and life chaos [13,31,34]. In this study, multivariate analysis showed that the association between concerns shown by others (emotional social support) and life chaos could not be considered as an independent association; therefore, adding an interaction without a main effect could not be considered. The statistically significant association found in bivariate analyses might be a confounded association due to the coherence between variables, i.e., concern shown by others and marital status (a common proxy to social support) [4]. As the association was eliminated in the multivariable model, emotional social support was not considered as a factor influencing life chaos. This finding was contradictory to a recent investigation that found a statistically significant association between social support and life chaos among methamphetamine male users who have sex with men (MSM) during transactional sexual encounters [31]. Another study concluded that life chaos was higher with low social support among HIV-infected persons [3]. A plausible explanation for the failure to determine an association between social support and life chaos is that the current study sample, which mainly constituted younger-aged men, might have had larger social networks through the emergence of cyber-technology, which offers virtual interconnectedness through online social networking activities, facilitates social support, and promotes rehabilitation. 

### 4.3. What Does This Study Add?

The current study was “hypothesis generating”, adding further evidence to pre-existing data to determine factors associated with “cluttered lives” among populations across different strata. The revolution of planetary health, which focuses on the effects of environment on human health and well-being, has catalyzed newer, modifiable risk factors as potential health threats. One such factor is life chaos and its impact on human physical and mental well-being. While preliminary efforts exploring life chaos in the European subcontinent showed mixed variations [3,4,10,29,31], the current study—the first in Asia from the Malaysian perspective—opens new vista for further exploration. Being a multiethnic region, the authors attempted to preliminarily explore associations between ethnicity and perceived life chaos. Data from the Malaysian National Cardiovascular Disease (NCVD) Database Registry found that ethnic Malays had the highest prevalence of STEMI, while the prevalence of NSTEMI was the highest among the ethnic Indians [35]. Previous studies have postulated that ethnic Indians with other disease comorbidities have higher scores of perceived psychological distress, anxiety, and depression [20,36]. A plausible explanation for this is that minority ethnic Indians are more vulnerable to be exposed to a variety of psychosocial stressors, such as high socio-economic constraints, poor education, and perceived discrimination [36], which could subsequently lead to elevated life chaos among this minority ethnic group. In this study, we found that ethnic Indians with MI had higher life chaos score in comparison to ethnic Malays and Chinese; however, these associations were not statistically significant. It is important to study the measure of life chaos as a potential new psychological factor in our setting given the alarming situation that mental health problems are projected to increase substantially in most developing countries, especially Malaysia, within the next decade [12]. 

### 4.4. Study Limitations

The cross-sectional nature of the current study could not establish temporality between covariates. The relatively small sample size from a single hospital limited the generalizability of the study findings. Although our primary outcome measure was novel, certain demographic variables that were hypothesized to be associated with life chaos as shown in previous literature were not proven, for example, ethnicity. These inconsistencies may be due to differences in the disease states (MI versus HIV), the environment where the studies were conducted (Asian versus European), recruitment strategies (clinic-based recruitment versus mobile outreach recruitment), population being studied (post-MI survivors versus HIV-infected persons or people with risky sexual behaviors), and differences in study designs (quantitative versus qualitative approach). It could be postulated that the religious views of patients—and, as a consequence, their attitude to illness and suffering—could be a predictor to influence patient behavior post-MI. As the current study attempted to explore the associations between ethnicity and life chaos, future studies should incorporate religiosity within multiethnic Malaysia to offset current limitations that missed a potentially untested variable, i.e., religiosity could be attributed as a coping mechanism or triggering factor of chaotic lifestyles in post-MI patients. Most literature have highlighted pre-existing psychological attributes like chronic stress to be associated with the development of cardiovascular disease and, in the case of particular types of stresses such as job and marital strain, recurrent adverse events is possible after acute MI. Psychological attributes like anxiety is a response that is likely to occur following a major cardiac event, but in its extreme form, significant distress, poor function, and detrimental outcomes may occur. Arnold and colleagues [37] found that psychological attributes like moderate to high perceived stress at the time of an acute MI is associated with adverse long-term outcomes. Thus, it would be crucial to explore if life chaos—a novel psychological attribute evaluated in this study—would cause serious adverse outcomes in its extreme form, leading to premature deaths or reinfarctions. As the current cross-sectional nature of the study was not powered to find such temporality between these factors, future prospective cohort studies are required to explore such possibilities.

## 5. Conclusions

The current study has shown the prevalence of life chaos and its potential correlates among post-MI survivors. High chaos was associated with demographic (younger age), socio-economic (low household income, perceived financial insecurity), and health-related (poor health status, ≥2 disease comorbidities) factors. Identifying chaotic lifestyles among individuals at an early stage is important to prompt caregivers and policy makers to implement and initiate potential life-coping skills to prevent “disorganized” and “disordered” lives. Robust methodological study designs, such as prospective cohorts or meta-analytic studies, are warranted to determine temporality between covariates in future studies.

## Figures and Tables

**Table 1:** Sample characteristics (*n* = 242).

Characteristics	*n*	(%)
Gender		
Men	208	86.0
Women	34	14.0
Age (years)		
18–59	156	64.5
≥60	86	35.5
Ethnicity		
Malay	125	51.7
Chinese	48	19.8
Indian	69	28.5
Marital status		
Single	9	3.7
Married	208	86.0
Divorced/Separated/Widowed	25	10.3
Household income (MYR) * (*n* = 236)		
≤1000	48	20.3
1001–3000	143	60.6
≥3001	45	19.1
Current employment status		
Working	110	45.5
Not working	132	54.5
Perceived financial insecurity		
No	81	33.5
Yes	161	66.5

* 1 MYR = USD 0.23 at the time of the study.

**Table 2:** Respondents health attributes and social support (*n* = 242).

Characteristics	*n*	(%)
Perceived general health status		
Good	177	73.1
Poor	65	26.9
Have diabetes		
No	127	52.5
Yes	115	47.5
Have hypertension		
No	78	32.2
Yes	164	67.8
Have hypercholesterolemia		
No	90	37.2
Yes	152	62.8
No. of comorbidities		
<2	88	36.4
≥2	154	63.6
Symptoms of heart failure		
No	203	83.9
Yes	39	16.1
Have atrial fibrillation		
No	232	95.9
Yes	10	4.1
Instrumental social support		
Very easy/easy	128	52.9
Possible	68	28.1
Difficult	46	19.0
Other people’s concern		
A lot	110	45.5
Some	86	35.5
None/little/uncertain	46	19.0
People to count on		
None/1–2 people	89	36.8
3–5 people	90	37.2
>5 people	63	26.0
Perceived life chaos score		
Low	114	47.1
High	128	52.9

**Table 3:** Association between sample characteristics and life chaos.

Characteristics	Life Chaos	
	Mean (SD)	*p*-Value
Gender		
Men	15.0 (5.2)	
Women	15.3 (5.5)	0.740
Age (years)		
18–59	15.7 (5.4)	
≥60	13.8 (4.8)	0.005
Ethnicity		
Malay	15.1 (5.2)	
Chinese	13.6 (4.5)	
Indian	15.7 (5.5)	0.100
Marital status		
Single	15.7 (6.0)	
Married	15.0 (5.3)	
Divorced/Separated/Widowed	15.0 (5.2)	0.935
Household income (MYR)		
≤1000	16.7 (6.1)	
1001–3000	14.8 (5.1)	
≥3001	13.9 (4.3)	0.027
Current employment status		
Working	14.7 (5.3)	
Not working	15.3 (5.2)	0.404
Perceived financial insecurity		
No	13.2 (4.9)	
Yes	16.0 (5.2)	<0.001

**Table 4:** Association between health attributes and social support with life chaos.

Characteristics	Life Chaos	
	Mean (SD)	*p*-Value
General health		
Good	14.2 (4.8)	
Poor	17.3 (5.7)	<0.001
Have diabetes		
No	14.3 (5.1)	
Yes	15.8 (5.3)	0.029
Have hypertension		
No	14.2 (5.2)	
Yes	15.5 (5.3)	0.072
Have hypercholesterolemia		
No	14.5 (5.6)	
Yes	15.4 (5.1)	0.205
No. of comorbidities		
<2	13.8 (5.2)	
≥2	15.8 (5.2)	0.005
Symptoms of heart failure		
No	15.0 (5.3)	
Yes	15.2 (5.1)	0.937
Have atrial fibrillation		
No	14.9 (5.2)	
Yes	17.0 (4.5)	0.235
Instrumental social support		
Very easy/easy	14.5 (5.0)	
Possible	15.8 (5.5)	
Difficult	15.5 (5.7)	0.198
Other people’s concern		
A lot	14.2 (4.9)	
Some	15.4 (5.6)	
None/little/uncertain	16.5 (5.0)	0.029
People to count on		
None/1–2 people	15.3 (5.4)	
3–5	15.3 (5.3)	
>5	14.3 (4.9)	0.395

**Table 5:** Factors associated with life chaos among post-myocardial infarction (MI) survivors by multiple linear regression analysis.

Life Chaos						
	B	SE	Beta	*p*-Value	95% CI	
					Lower	Upper
Age (18–59 years)	2.0	0.7	0.2	0.004	0.6	3.3
Household income (≤MYR 1000)	1.8	0.8	0.1	0.026	0.2	3.4
Perceived financial insecurity	1.9	0.7	0.2	0.006	0.6	3.3
Perceived poor health status	1.9	0.8	0.2	0.013	0.4	3.4
≥2 comorbidities	1.7	0.7	0.2	0.014	0.3	3.0

**Notes:** The reference group is ≥60 years for age; ≥MYR 3001 for household income; “No” for perceived financial insecurity; “Good” for perceived health status; <2 for number of comorbidities. B: unstandardized coefficients, SE: standard error, Beta: standardized coefficients CI: confidence intervals. Variables entered: all significant variables in bivariate analyses.
